# Emergency cesarean section under general anesthesia using remimazolam in a pregnant woman with Fontan circulation: Case report with literature review

**DOI:** 10.1007/s00101-025-01507-8

**Published:** 2025-02-13

**Authors:** Sun Woo Nam, Haesun Jung, Jiwon Han

**Affiliations:** Department of Anesthesiology and Pain Medicine, Chung-Ang University College of Medicine, 84, Heukseok-ro, Dongjak-gu, Seoul, Korea (Republic of)

## Medical history

A 32-year-old woman (weight 53 kg, height 164.7 cm, para 1) with a gestational age of 32 weeks and 3 days was admitted to the emergency department due to premature rupture of membranes. She was diagnosed with congenital heart disease (CHD), a double-outlet right ventricle, complete atrioventricular septum defect, pulmonary atresia and patent ductus arteriosus. Her surgical history included fenestrated Fontan operation at 2 years of age and fenestration closure at 3 years of age. Since then she was under periodic observation without any symptom aggravation at the center where she had undergone heart surgery. During pregnancy she had been taking 100 mg aspirin, which was planned to be discontinued after 36 weeks. Regarding her pregnancy-related care, she was followed-up at a women’s hospital near her home but was transferred to our center due to bed availability in the neonatal intensive care unit (NICU).

## Observations

Preoperative electrocardiogram showed sinus rhythm (heart rate 73 bpm), incomplete right bundle branch block, left anterior fascicular block and right ventricular hypertrophy. Echocardiography showed a single ventricle with preserved contractility (ejection fraction 56.1%), mild to moderate mitral valve regurgitation and trivial aortic valve regurgitation. Laboratory findings were within normal ranges except mild anemia (hemoglobin 10.4 g/dl) and mild thrombocytopenia (platelet count 111,000/μl). Arterial blood gas analysis (ABGA) showed pO_2_ 78.3 mm Hg and paCO_2_ 25.8 mm Hg in room air. The fetus was in a breech presentation and estimated fetal weight was 1567 g.

The patient was treated with atosiban (a competitive antagonist of oxytocin) infusion for tocolysis and two doses of betamethasone (12 mg, intramuscular, 24 h apart) for fetal lung maturation were administered. With uterine contractions not subsiding and a non-stress test showing intermittent minimal fetal heart rate variability, emergency cesarean section was decided at gestational age of 32 weeks and 5 days. After deciding on the surgery, preoperative consultations were made with a cardiologist, cardiovascular surgeon, and pediatrician to prepare for intraoperative events and postoperative care.

## Treatment and course

Considering the risk of epidural hematoma following catheterization or needling owing to continued aspirin use and thrombocytopenia and rapid decrease in systemic vascular resistance (SVR) that could cause hemodynamic instability, general anesthesia was chosen over regional anesthesia. The patient was intraoperatively monitored using pulse oximetry, electrocardiography, noninvasive blood pressure measurement and bispectral index (BIS, A‑2000 XP; Aspect Medical System, Newton, MA, USA). For continuous blood pressure monitoring, the radial artery was catheterized. With a pediatrician on standby, after applying a surgical drape to the patient, remimazolam was infused at 6 mg/kg/h and remifentanil at 3 ng/mL using target-controlled infusion (Minto model; Orchestra®; Fresenius-Vial, Brezins, France). Upon confirming that the BIS value dropped below 60, rocuronium (50 mg) was administered, manual ventilation was initiated and surgery was begun immediately after endotracheal intubation. Anesthesia was maintained with remimazolam at 1–2 mg/kg/h and remifentanil at 1–2 ng/ml with target-controlled infusion. During surgery a tidal volume of 325–375 ml without positive end-expiratory pressure (PEEP) was administered, which resulted in a mean airway pressure of 4–5 cmH_2_O, and the normal range of end tidal CO_2_ (EtCO_2_) was achieved with a respiratory rate of 7–10 breaths/min. The baby was delivered 2 min after surgery initiation and cried immediately (1-min APGAR score of 7, activity: 1, pulse: 2, grimace: 2, appearance: 1, respiration: 1). After standard neonatal care including tactile stimulation, drying and warming and clearing the airway, approximately 30 s of positive pressure ventilation was performed. At 5 min after delivery the baby’s respiratory status improved and the APGAR score was 9 (activity: 1, pulse: 2, grimace: 2, appearance: 2, respiration: 2). The newborn, weighing 1746 g, was subsequently transferred to the neonatal intensive care unit (NICU). After placental delivery carbetocin (0.1 mg) mixed with 0.9% NaCl (100 ml) was administered. The surgery was completed after confirming adequate hemostasis and uterine contraction. For recovery after general anesthesia sugammadex (200 mg) was administered with a train-of-four count of 1 (NMT Mechanosensor; GE Healthcare, Berlin, Germany). In addition, flumazenil (0.2 mg) was administered to ensure rapid reversal of the effects of remimazolam and to help prevent possible hypoventilation. The endotracheal tube was removed after confirming that the patient’s BIS value was > 90 and the train-of-four ratio was > 98%. The patient was hemodynamically stable and no inotropics or vasopressors were required. Total surgical time was 23 min and anesthesia time was 45 min. Intraoperatively, 600 ml of crystalloid was administered, estimated blood loss was 400 ml and urine output was 150 ml. The patient was transferred to the intensive care unit with 5 l/min of oxygen applied via a facial mask.

During the immediate postoperative period, a cardiologist provided patient care. Postoperative ABGA showed pO_2_ 197.1 mm Hg and paCO_2_ 26.1 mm Hg with 4 l/min of oxygen applied via a facial mask, and oxygen supplementation was slowly tapered with target SpO_2_ of ≥ 95%. Postoperatively, the patient’s hemoglobin level dropped to 7.8 g/dL; hence, two packs of red blood cells were transfused and 15 ml ferric hydroxide (300 mg iron) was administered. On postoperative day 1 the patient was transferred to a general ward with hemoglobin 10.4 g/dl and stable vital signs without oxygen supplementation. The patient’s chest X‑ray, electrocardiogram, and cardiac enzymes were checked daily and showed no abnormalities. The patient was transferred to the obstetrics department on the 3rd postoperative day and discharged on the 7th postoperative day without complications.

The baby maintained SpO_2_ above 95% on room air in the NICU and ventilator care was unnecessary. An echocardiogram performed on the 5th day after birth showed normal results and a brain ultrasound performed on the 7th day after birth showed a heterogeneous periventricular white matter echo and no germinal matrix or intraventricular hemorrhage. Additionally, a screening test for congenital metabolic abnormalities in newborns performed on the 7th day after birth showed normal results. The baby received NICU care for phototherapy owing to neonatal jaundice and was discharged on the 30th day after birth.

## Discussion

To our knowledge, this is the first case report of cesarean section under general anesthesia using remimazolam in a patient with Fontan circulation. Herein, general anesthesia was performed using remimazolam, remifentanil, and rocuronium and the mother remained hemodynamically stable during surgery. She recovered appropriately after administration of flumazenil and sugammadex. The newborn was also stable without complications.

### Fontan physiology

**Fig. 1:**
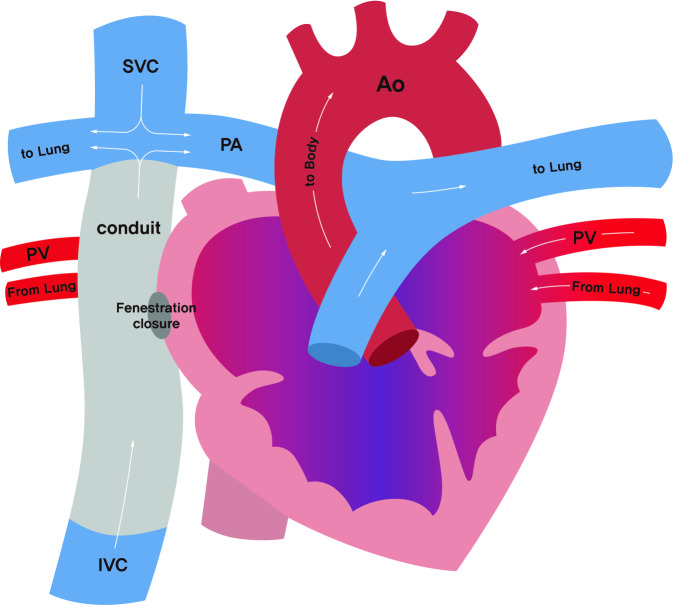
Diagram showing the Fontan procedure for patients with double-outlet right ventricle. *Ao* Aorta, *IVC* Inferior Vena Cava, *PA* Pulmonary artery, *PV* Pulmonary vein, *SVC* Superior Vena Cava

Fontan surgery is performed on patients with single ventricular physiology, including tricuspid atresia, pulmonary atresia with intact ventricular septum, double-outlet right ventricle, complete atrioventricular septal defects, and hypoplastic left heart syndrome. Fontan surgery aims to connect the caval venous return directly to pulmonary circulation without involving the ventricle and allow the single ventricle to handle only the systemic circulation (Fig. 1). The surgical technique has developed over time from atriopulmonary to cavopulmonary (lateral tunnel or extracardiac) to avoid right atrium (RA) involvement responsible for RA dilatation and complications including atrial arrhythmia and thrombosis. A Fontan circulation is usually achieved through staged operations, such as systemic pulmonary shunt and Glenn operation, to avoid the relatively high pulmonary vascular resistance (PVR) of the neonatal period and to enable the cardiopulmonary system to gradually adapt. Some patients with suboptimal PVR may need a fenestration between the conduit and atrium to reduce caval venous pressure at the expense of persistent right to left shunt and slight desaturation. The Fontan circulation resolves cyanosis and ventricular overloading, making it possible for most patients to survive until adulthood; however, these patients have a significantly decreased cardiac reserve and often suffer long-term morbidity and mortality such as arrhythmia, thrombosis, heart failure and protein-losing enteropathy [1]. The pulmonary blood flow in Fontan circulation is nonpulsatile and the driving force is achieved by a gradient of central venous pressure (CVP) and pulmonary artery occlusion pressure. Therefore, to maintain pulmonary flow, PVR must be kept low and hypoxia, hypercapnia, acidosis and excessive airway pressure that cause an increase in PVR must be avoided. Because Fontan circulation is highly preload-dependent, hypovolemia is poorly tolerated and must be strictly avoided. To maintain adequate ventricular filling and cardiac output, atrial arrhythmia and rapid ventricular response can be fatal, therefore strict maintenance of sinus rhythm is recommended [2].

#### Cardiovascular complications and obstetric outcomes in mothers with Fontan circulation

Various cardiovascular complications can occur due to increased circulatory loading and the hypercoagulable state following pregnancy. The most frequent complication was reported to be supraventricular arrhythmia (8.4%), followed by heart failure (3.9%) and systemic embolism (1.7%) [3]. Obstetric outcomes in parturients with Fontan physiology were relatively poor (pregnancy loss rate 69%; mostly spontaneous miscarriages and some iatrogenic terminations). Reportedly, 59% of live births were premature deliveries and the cesarean section rate was 57%, which was much higher than that in the general parturient population [3]. Additionally, the postpartum hemorrhage risk also increased (14%), which is considered to be due to increased venous pressure, arteriovenous malformation in the uteroplacental circulation, use of anticoagulants and coagulation factor abnormalities. Nevertheless, the mortality and fatal complications have been relatively rarely reported [3]. Fontan patients are recommended to undergo frequent surveillance during pregnancy (i.e., monthly) and in the first weeks after delivery. Furthermore, pregnant Fontan patients are at increased risk of thromboembolic complications and prophylactic anticoagulation treatment is recommended (class IIa), balanced with the risk of bleeding. Pregnancy is not recommended in patients with saturation < 85%, depressed ventricular function, moderate to severe aortic valve regurgitation, refractory arrhythmia, or protein-losing enteropathy (class III) [4].

#### Obstetric anesthesia for Fontan physiology

Ideally, in moderate or high-risk parturients, delivery is performed at a tertiary center by an experienced cardio-obstetric anesthesia team [5]. Unless the patients’ cardiac function is decompensated, the decision between vaginal delivery and cesarean delivery can be an obstetric decision. Vaginal delivery with slow titration of epidural labor analgesia is preferred to reduce hemorrhage, thrombosis and infection, although Valsalva maneuvers in the 2nd stage of labor can burden the cardiovascular system owing to increased PVR [2].

If cesarean section is chosen, regional anesthesia may be more advantageous than general anesthesia, which inevitably requires mechanical ventilation owing to an increase in PVR; however, spinal anesthesia is not recommended as it causes an abrupt decrease in SVR and venous return, and gradual epidural anesthesia is generally mostly recommended. Nevertheless, when choosing anesthetic methods, the patients’ platelet and coagulation status along with prophylactic anticoagulation regimen should be checked and the risks and benefits of neuraxial anesthesia should be assessed [6, 7].

Only a few case reports and case series exist on anesthesia for cesarean section in Fontan patients (Table 1). Most cases reported using epidural anesthesia, with multiple divided doses of 2% lidocaine and 0.75% ropivacaine. Meanwhile, for general anesthesia induction, various induction agents including propofol, etomidate, thiopental were used; however, a study recommended agents such as etomidate or ketamine that provide more hemodynamic stability [2]. Using high-concentration inhalation anesthetics over 1.5 times the minimum alveolar concentration should be avoided because of side effects such as arrhythmia and postpartum hemorrhage from decreased myometrial contractility. Also, special attention must be paid to long-acting opioids and residual neuromuscular blockade that could cause respiratory depression. During mechanical ventilation, to achieve adequate pulmonary blood flow and low PVR, low tidal volume (5–6 ml/kg), low PEEP and low respiratory rates with short inspiratory times are recommended. Maintaining normocarbia is desirable; both severe hypercapnia and excessive hyperventilation that could cause increased intrathoracic pressures should be avoided [1]. Reduced venous return due to hemorrhage or aortocaval compression is poorly tolerated. The use of CVP monitoring and vasoactive agents through a central line may be helpful in decompensated cases. In Fontan circulation, CVP directly indicates the mean pulmonary arterial pressure, and 10–15 mm Hg is known to be the optimal value. In patients with residual right to left shunts including fenestration, caution is required regarding air embolism to the intravenous line. As judicious attention to ventricular function and fluid replacement is required in the postpartum period, intensive care unit admission can generally be considered for about 24–48 h postoperatively.

**Table 1:** Case reports regarding anesthesia for cesarean section in Fontan patients

Study (year)	Emergency/elective	Gestational age (weeks)	Anesthetic method	Anesthetic regimen	Events
Carp et al. (1994) [14]	Emergency	30	Epidural	2% lidocaine 25 ml, sufentanil 20 μg (5-ml increments over 30 min)	–
Komori et al. (1999) [15]	Elective	38	General	I: thiopental 250 mg, fentanyl 100 μg	PPH requiring transfusion
Eid et al. (2005) [16]	Emergency	26	General	I: propofol 150 mg M: isoflurane, N_2_O	–
	Elective	36	Epidural	2% lidocaine 20 ml (4 times of 5‑ml boluses), fentanyl 100 μg	–
Ioscovich et al. (2006) [17]	Emergency	36	Epidural	2% lidocaine 20 ml (5-ml increments at 3‑min intervals)	Labor analgesia → surgical conversion
Grim et al. (2011) [18]	Emergency	33	General	I: etomidate 0.3 mg/kg M: sevoflurane 1.7% vol	Delayed extubation due to pseudocholinesterase deficiency
Mathney et al. (2015) [19]	Elective	36	Epidural	2% lidocaine 16 ml, epinephrine 1:200,000	–
Chiaghana et al. (2016) [20]	Elective	36	Continuous spinal	–	Neuraxial hematoma
Wu et al. (2019) [21]	Emergency	28	Epidural	2% lidocaine 5 ml, 0.75% ropivacaine 10 ml	–
Saito et al. (2019) [22]	Emergency	28	General	I: propofol 120 mg M: propofol 5 mg/kg/h	PPH requiring reoperation
Saito et al. (2020) [23]	Elective	38	Combined spinal-epidural	Spinal: hyperbaric bupivacaine 5 mg, fentanyl 15 μg Epidural: 2% lidocaine 10 ml	–
Ohsugi et al. (2023) [24]	Emergency	26	General	I: midazolam 5 mg, remifentanil 0.1 μg/kg/min, fentanyl 150 μg M: sevoflurane 1.5% vol, remifentanil, intermittent fentanyl	PPH due to placenta increta; intra-aortic balloon applied

*PPH* postpartum hemorrhage*, I,* induction agent of anesthesia; *M* maintenance agent of anesthesia

Caution may be required in tocolytic and uterotonic use, although there is not much data regarding their use in Fontan physiology. Betamimetic agents such as ritodrine are contraindicated in patients with pulmonary hypertension, right to left shunt, aortic/mitral/pulmonary stenosis, hypertrophic cardiomyopathy and coarctation of the aorta. Moreover, betamimetic agents can cause pulmonary edema, myocardial ischemia-like symptoms, arrhythmia, atrioventricular block and hypokalemia and are typically avoided in patients with cardiac disease [8]. Other mechanism of tocolytics, ergot alkaloids (e.g.,. methylergonovine), or prostaglandin F2alpha analogs (carboprost) should be avoided in patients with Fontan physiology because they could induce pulmonary vasoconstriction [4]; however, as these patients are sensitive to hypovolemia due to postpartum hemorrhage, the use of uterotonics is important and slow injection of oxytocin analogues with meticulous hemodynamic monitoring or prostaglandin E analogs such as misoprostol and sulprostone, is relatively safe [5].

### Remimazolam in obstetric anesthesia

To date, only a few retrospective analyses and case reports exist on remimazolam use in obstetric anesthesia. Reportedly, there was no difference in the additional use of uterotonics or estimated blood loss and the length of stay was reportedly less in women who were anesthetized with remimazolam compared to those with propofol [9]; however, considering the lack of data on the effects of remimazolam on the fetus, induction and sevoflurane were maintained with thiopental in both groups, and propofol or remimazolam was maintained only after the fetus was delivered. A previous case report showed that emergency cesarean section was safely performed with the patient under general anesthesia using remimazolam in a female with heart failure due to infective endocarditis [10].

Although not in obstetric anesthesia, studies have shown that remimazolam maintains a higher cardiac output than propofol [11]; however, its effect on SVR seems controversial [11, 12]. Also, remimazolam does not cause compensatory tachycardia like thiopental does and has minimal effects on PVR [13]. Moreover, rapid reversal with flumazenil may reduce hypoventilation after recovery from anesthesia that could increase PVR. Considering the current findings, remimazolam use in pregnant women with cardiovascular diseases seems reasonable; however, as the possibility of placental transfer of remimazolam cannot be ruled out, further studies on its effect on the newborn’s condition after delivery are warranted.

### Limitations

In our case the patient’s cardiac function was well-preserved at the time of delivery; hence, the findings may not be generalizable to mothers with decompensated cardiac function.

## Conclusion

Remimazolam can be considered as a reasonable choice when performing a cesarean section with the patient under general anesthesia in women with Fontan circulation. Further studies on its impact on mothers and newborns are warranted.

## Declarations

This case report was approved by the Institutional Review Board (IRB) of Chung-Ang University Gwangmyeong Hospital (IRB approval no. 2407-169-082, approval date: 17 July 2024). This study was conducted in accordance with the Declaration of Helsinki and its later amendments. Informed consent was waived by the IRB.
